# Perception of Childhood Obesity and Support for Prevention Policies among Latinos and Whites

**DOI:** 10.1155/2014/328276

**Published:** 2014-06-19

**Authors:** Douglas M. Puricelli Perin, Leah Frerichs, Sergio Costa, Amelie G. Ramirez, Terry T.-K. Huang

**Affiliations:** ^1^Center for Global Health and Development, College of Public Health, University of Nebraska Medical Center, Omaha, NE 68198-4341, USA; ^2^Department of Health Promotion and Social and Behavioral Health, College of Public Health, University of Nebraska Medical Center, 984365 Nebraska Medical Center, Omaha, NE 68198-4365, USA; ^3^University of Nebraska Medical Center, Omaha, NE 68198-4355, USA; ^4^Institute for Health Promotion Research, University of Texas Health Science Center, San Antonio, TX 78229, USA

## Abstract

A cross-sectional survey was administered to Latino and White residents of Omaha, NE, to assess perception of the childhood obesity problem, attribution of responsibility, and support for obesity-related policies. The sample included 40.8% (*n* = 271) Latinos and 59.2% (*n* = 393) Whites. Among Latinos, 25% did not see childhood obesity as a problem, compared to 6% of Whites (*P* < 0.001). This difference persisted after adjusting for age, gender, and education level (odds ratio (OR) 2.10, 95% confidence interval (CI) 1.07–4.14). Latinos were more likely to agree that government was responsible for addressing childhood obesity compared to Whites (OR 2.81, 95% CI 1.82–4.35). Higher support for policy interventions was observed among individuals who perceived childhood obesity as a big problem compared to those who did not, independent of race, sex, age, or education level. The relationship between support for tax-based policies and perception of the childhood obesity problem was mainly evident among Latinos rather than Whites. Despite city-wide efforts to address obesity, differential penetration in community subgroups appears evident. There is room to further engage Latinos in the cause of obesity. Deepening community awareness about the consequences and complexity of childhood obesity can lead to stronger support for childhood obesity policy interventions.

## 1. Introduction

Recent trends of childhood obesity suggest that there is a leveling but persistently high prevalence in the US [1]. National averages, however, may mask the potential for widening disparities in obesity prevalence across population groups. For example, Mexican American adults have shown the largest increase in obesity rates in the last decade [2], Mexican American boys continue to have the highest prevalence of obesity rates among all US children [2], and the proportion of reversals in obesity between 2008 and 2011 among Mexican American children is significantly lower than that of White children [3]. These nuanced trends may in part be the result of differential penetration of community-wide efforts in population subgroups, as residential segregation and language and cultural barriers remain dominant in US cities. In Omaha, NE, although there have been substantial city-wide efforts around healthy living over the past decade, it is unclear whether Latino residents have benefited from or responded to these efforts in the same way as the majority of White population.

Understanding public perceptions of childhood obesity is an important way to gauge social change and could directly inform strategies for adopting or implementing obesity prevention policies [4]. There is limited information on public opinions of childhood obesity. According to a recent survey, 73% of the US voting population considered obesity prevention as a political priority, with 58% reporting it as a very important priority for the government. In addition, 62% of the respondents agreed that saving money should not be a concern when investing in solutions for this problem and 61% saw that the problem could be solved within a generation if properly managed [5]. That study, however, did not address specific policy options for childhood obesity or examine the data by race. Public perception was further investigated in another national household survey with questions about perception of the severity of childhood obesity and support for specific intervention strategies [6, 7]. It is not clear from this study, however, how the perception of childhood obesity relates to support for policies. Although the results suggested that perception of childhood obesity as a serious problem might lead to stronger support for specific policies, this relationship was not examined directly. Moreover, Latinos were not specifically studied.

The aims of the current study were to (1) compare the perception of childhood obesity as a problem, attribution of responsibility, and support for obesity policies between Latino and White residents in Omaha, NE, and (2) determine the association of support for obesity policies with perception of childhood obesity as a problem.

## 2. Materials and Methods

As part of a larger community initiative focused on Latinos in Omaha, a cross-sectional survey of adults living in Douglas County, Nebraska, was conducted. Douglas County is home to the state's largest metropolitan area of Omaha, with 524,861 residents (73% White, 11% Latino) [8]. The southeast sector of Omaha has been settled by a significant number of Latinos in the past two decades. Consequently, the Latino community has more than doubled and, in certain areas, they represent between 32% and 55% of the population [8].

The survey instrument was developed to assess perceptions of the childhood obesity problem, parties responsible, and support for select policy strategies to address the problem. The questions were developed and adapted using an existing survey tool from the literature [7]. Participants were asked how big of a problem they considered childhood obesity to be in order to assess community perception of the problem. Locus of responsibility was examined by asking participants how much they agreed or disagreed with the role of caregivers and parents, children, and government in addressing the childhood obesity problem. Support for specific policies was assessed in two questions—“In response to each of the following policies, please indicate how strongly you agree or disagree that each should be implemented in your community: (a) tax junk foods and use the funds gained to support the production and distribution of healthy food; (b) ban fast food vendors in schools; (c) establish and mandate school lunch nutrition standards; (d) establish and mandate physical fitness education in schools; (e) tax soda/pop”—and—“How likely would you be to pay increased taxes for the following items? (a) Better public transportation; (b) increased opportunities for physical activity (i.e. parks, walking trails, and playground equipment); (c) better housing for the poor; (d) more nutritious/healthier school lunches.”

The survey was conducted online and in person, in both English and Spanish, from March to May 2011, using convenience and snowball sampling techniques at a range of locations and venues. The online survey was distributed via multiple Greater Omaha and neighborhood-specific community and organizational email distribution lists as well as advertised via Facebook. Informational flyers that contained the online survey link were also distributed during community meetings and events. Hard copies were administered at multiple public events throughout Omaha, including two health fairs, a Cinco de Mayo festival, and two community-based coalition meetings. In addition, participants were recruited in-person at three Department of Motor Vehicle locations, five public library branches, and the waiting rooms of four community-based primary care clinics. Among Latino respondents, 46.2% of the surveys were completed in English and 53.8% in Spanish (20.3% of the total sample).

Because of small numbers, surveys from races other than Latino and White were excluded from analysis. Descriptive statistics were used to generate overall sample characteristics. For ease of interpretation in this particular paper, Likert-scale responses to policy support questions were recoded to dichotomous outcome variables (e.g., “strongly agree and agree” versus “neutral, disagree, and strongly disagree”). Crude differences between Latinos and Whites were analyzed by chi-square tests. Logistic regression was used to model policy support on the perception of childhood obesity as a problem (“not a big problem,” “neutral,” and “a big problem”), race (Latino and White), sex, age, and education. Regression analysis was also stratified by race. All analysis was conducted using SPSS v.9.1 (Cary, NC) with a two-sided alpha level of 0.05.

## 3. Results

### 3.1. Sample Characteristics

A total of 664 survey responses were used for this analysis. Latinos represented 40.8% (*n* = 271) and Whites represented 59.2% (*n* = 393) of the respondents. Sample characteristics are shown in Table 1. The sample was comprised by more females than males among both Latinos and Whites. Latinos were younger and had lower educational level than Whites (*P* < 0.001). More than one-third of Latinos in the sample did not graduate from high school (35.6%) and only 13.7% had a bachelor's or higher degree, compared to over 60% of Whites. Both racial groups were more educated than the average for each group in Omaha, according to the American Community Survey data [8].

### 3.2. Perceptions of the Childhood Obesity Problem

Latinos had a significantly lower level of recognition of the importance of the childhood obesity problem, compared to Whites. Both positive and negative attitude statements were included to confirm survey item validity. Among Latinos, 78.2% identified childhood obesity as a “big or very big” problem, compared to 91.3% of Whites who said so (*P* < 0.001). Conversely, 25% of Latinos compared to 6% of Whites either agreed or strongly agreed with the statement, “I do not believe there is a childhood obesity problem” (*P* < 0.001). This difference persisted after adjusting for age, sex, and education level (odds ratio (OR) 2.10, 95% confidence interval (CI) 1.07–4.14).

### 3.3. Responsibility for Addressing Childhood Obesity

**Figure 1:**
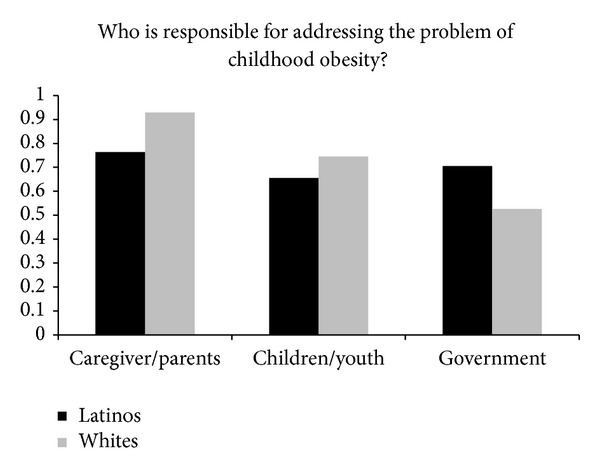
Perception of responsibility in addressing childhood obesity.

Survey respondents were asked to rate how much they agreed or disagreed with parents, children, or the government as the responsible party for the problem of childhood obesity (Figure 1). Among Latinos, 76.4% indicated that caregivers and parents were responsible, compared to 92.9% of Whites. After adjusting for age, sex, and education level, this difference between Latinos and Whites over parents' or caregivers' responsibility remained significant (OR 0.48, 95% CI 0.26–0.87). Regarding the role of children, 65.6% of Latinos agreed that it was their responsibility compared to 74.5% of Whites, but this difference became nonsignificant after adjusting for age, sex, and education level (OR 0.82, 95% CI 0.53–1.27). Finally, 70.5% of Latinos agreed that it was the government's responsibility to address childhood obesity, compared to 52.6% of Whites, and the association remained significant after accounting for covariates (OR 2.81, 95% CI 1.82–4.35).

### 3.4. Support for Tax Interventions

Among Latinos, 48.1% were supportive of a tax on junk foods and using the added revenue to support the production and distribution of healthy foods compared to 39.9% of Whites. Independent of the perception of childhood obesity as a problem, age, sex, and education level, Latinos were more supportive than Whites of taxation of junk foods (OR 1.94, 95% CI 1.26–2.99). In addition, 80.6% of Latinos supported paying higher taxes for healthier school lunches compared to 71.4% of Whites; 74.2% of Latinos would pay more taxes if it were to increase opportunities for physical activities compared to 68.9% of Whites, and more Latinos supported taxes for improving housing for the poor (63.4%) and better public transportation (48.9%) versus Whites (47.4% and 46.7%, resp.). After adjusting for age, sex, education level, and perception of childhood obesity as a problem, the odds of higher support among Latinos compared to Whites remained significant for healthier school lunches (OR 2.69, 95% CI 1.56–4.62), increasing opportunities for physical activities (OR 2.56, 95% CI 1.55–4.24), improving housing for the poor (OR 2.59, 95% CI 1.67–4.02), and improving public transportation (OR 1.64, 95% CI 1.07–2.52).

### 3.5. Support for Policies in relation to Perception of Childhood Obesity as a Problem

**Figure 2:**
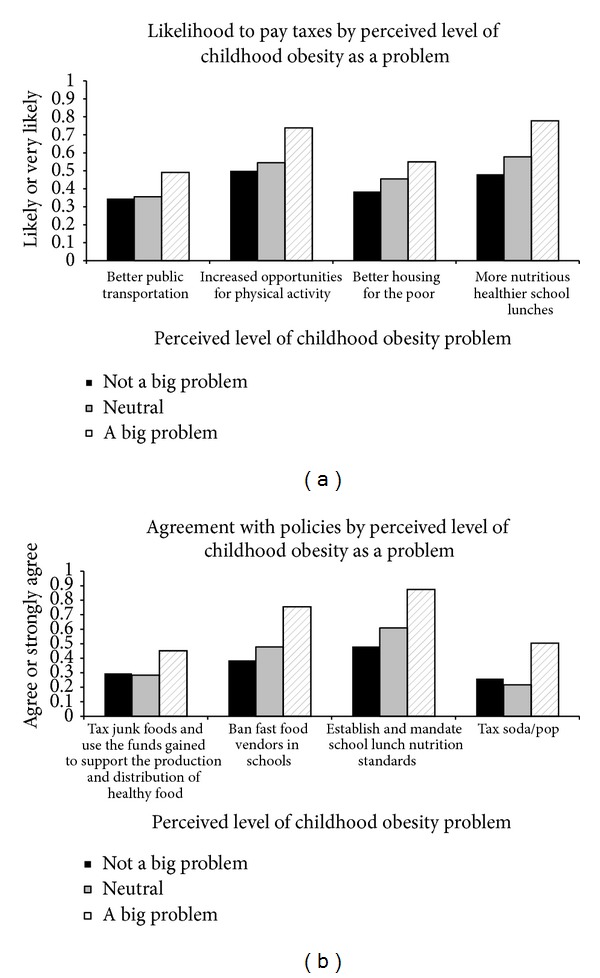
Support for interventions by perception of childhood obesity as a problem.

Higher support for policy and tax-based interventions was observed among individuals who perceived childhood obesity as a big problem compared to those who did not (Figure 2). School lunch nutrition standards were supported by 87.5% of individuals who saw childhood obesity as a big problem compared to 48.1% among those who did not. The ban of fast food vendors from schools was favored by 75.5% of those who recognized childhood obesity as a big problem compared to 38.5% among individuals who did not. Similar results were found for supporting taxation of junk foods and sugar-sweetened beverages and for increased opportunities for physical activity. After adjusting for race, age, sex, and education level, individuals who perceived childhood obesity as a big problem offered higher support for taxation of sugar-sweetened beverages (OR 3.18, 95% CI 1.22–8.27), school ban on fast food vendors (OR 4.61, 95% CI 1.90–11.21), school lunch nutrition standards (OR 6.54, 95% CI 2.69–15.89), healthier school lunches (OR 4.31, 95% CI 1.80–10.29), and increased opportunities for physical activity (OR 2.99, 95% CI 1.25–7.12) when compared to individuals who did not perceive it as being a big problem. Taxing junk food and paying increased taxes for better public transportation and housing for the poor were not significantly associated with perception of childhood obesity problem.

Table 2 shows race-stratified analysis of the association between support for policies and perception of childhood obesity as a problem. Independent of age, sex, and education level, significant association was observed among both Latinos and Whites who perceived childhood obesity as a big problem for school ban on fast food vendors and school lunch nutrition standards. More support for taxation of sugar-sweetened beverages was observed among Latinos (but not among Whites) who perceived childhood obesity as a problem compared to those who did not (OR 4.00, 95% CI 1.24–12.86) after adjusting for the other covariates. Similar pattern was seen in increasing taxes in order to provide better housing for the poor (Latino OR 2.98, 95% CI 1.04–8.55) and healthier school lunches (Latino OR 4.47, 95% CI 1.59–12.55). Tax on junk foods and raising taxes to improve public transportation and increase opportunities for physical activity were not significantly associated with perception of childhood obesity problem for either group.

## 4. Conclusions

Our findings have important implications for the continuing epidemic of health disparities and suggest the importance of similar inquiry in other communities in the US. Although four times as many Latinos did not recognize childhood obesity as a problem compared to Whites, Latinos were more favorable towards government and policy interventions than Whites. Interestingly, although a higher proportion of Whites recognized childhood obesity as an urgent problem compared to national statistics, a lower proportion of Whites in Omaha supported government intervention compared to the national mean [5]. Support for certain policy interventions was associated with recognition of childhood obesity as a problem. However, the relationship between perception of childhood obesity as a problem and support for tax-based interventions was mostly driven by Latinos. The finding reinforces that perception of childhood obesity as a problem is a legitimate construct, and that it is important to address when assessing policy interventions.

A few surprising findings emerged in this study. First, recognition of obesity as a big problem was associated with increased support for tax-based obesity prevention policies only among Latinos, not Whites. However, this relationship among Latinos only existed for taxing sodas and not junk food in general. Second, education rather than perception of obesity was a strong correlate of policy support among Whites but not Latinos, suggesting that different approaches to mobilizing Latinos and Whites may be needed. Third, although Latinos were more favorable toward paying increased taxes for healthy school nutrition with increasing recognition of the obesity problem, they did not necessarily support obtaining revenue through junk food taxes. This suggests a need for greater transparency and public communication about how governments would use food-based tax revenue. Finally, there was no significant relationship between obesity perception and support for physical activity-related policies, perhaps because much of the media discourse around obesity has been centered on food rather than physical activity.

Our study confirms previous findings [5] that Latinos were more likely than Whites to agree on the major role of government and policy change in addressing childhood obesity. Latinos register as Democrats by a 2 : 1 margin, and Democrats are usually more in favor than Republicans of a prominent role of government in providing services to the community. However, Latinos also are more willing to support candidates who have a meaningful agenda with respect to issues relevant to the Latino community independent of party affiliation [9], thus providing an opportunity to increase the political demand for healthy policies in both parties. Latinos play an increasingly important role in election outcomes. The continued increase in population size as well as active participation at the polls suggests increasing political power among Latinos throughout the country [9]. Thus, Latinos' perception of childhood obesity is of particular interest in view of policy design and implementation, as it may become decisive in the near future.

As the largest and fastest growing minority in the US [10], with a significant stake in childhood obesity prevention due to growing disparities, a redoubling of effort to work with the Latino community should be a priority in public health. However, in spite of the large increase in obesity research over the last two decades, very few studies have focused on the Latino population [11]. The lack of data on Latinos is an obstacle for the development and implementation of relevant prevention programs [12].

This study contrasts with the results of a 2011 survey conducted by the Public Policy Institute of California [13], where 98% of Latinos declared obesity to be a very serious or somewhat serious public health problem compared to 94% of Whites. Awareness among Latinos in California seems to be much higher when compared to the Omaha sample, perhaps due to the highly publicized community intervention initiatives from the California Endowment [14]. In addition, a national poll by Mott Children's Hospital at the University of Michigan found that slightly more Latino (44%) than White (37%) adults rated childhood obesity as the top concern on children's health [15]. These numbers are not directly comparable to this study because the Mott survey item included a long list of public health issues being ranked in the same question. Differences across surveys suggest that large variability of social attitudes may exist across the US. Although local contexts are often not captured in national polls, many obesity policies are debated and implemented at the local or state level. The lack of granular statistics at the local level is a major gap in surveillance. The California study did show that only 33% of Latinos reported that individuals and families were more responsible than government for addressing obesity compared to 52% of Whites, suggesting that the group differences found in our study may be generalizable to elsewhere in the US.

Study limitations include the cross-sectional nature impeding causal inference on the relationships observed and the use of convenience sampling, which restricts the generalizability of the results. Although selection bias may be an issue and unknown confounding factors cannot be ruled out due to the sampling method used, the fact that both groups were slightly more educated than the Omaha average in each group indicates that such bias is likely to be random between Latinos and Whites. It is arguable that group differences may in fact be wider between the two groups had the study included more participants from lower education levels, given that lower educated Whites tend to be more politically conservative and antigovernment [16]. Finally, the set of policy issues is by no means comprehensive in this study. However, the items were selected to represent both microlevel (e.g., school policies) and macrolevel (e.g., taxation) issues. These items serve as proxy indication of where Latino and White residents in the center of the country stand politically vis-à-vis the intersection of public health and public policy.

In spite of study limitations, to our knowledge, this is one of the first studies to document the perception of Latinos regarding childhood obesity and related policies. Of note, information from the middle of the country is particularly limited. This study also highlights a promising new research arena from a policy standpoint. There is significant room to increase Latino community's engagement in the childhood obesity debate. Substantial support exists among Latinos for policy interventions in public health, but many Latinos remain unaware of childhood obesity as an issue. Social marketing and public engagement efforts to raise community awareness may prove valuable to strengthen the support for specific policy strategies to address this problem. Because of Latinos' rising political clout in the US, Latinos may be a particularly important demographic for public health policy goals.

## Figures and Tables

**Table 1:** Sample characteristics.

	Latino (%) *N* = 271	White (%) *N* = 393	*P*-value
Age			
19–24	15.2	10.4	<0.001∗
25–34	35.3	28.8	
35–44	30.1	16.8	
45–54	13.8	18.6	
55–64	5.2	17.0	
65 and over	0.4	8.4	
Sex			
Female	67.4	65.6	0.65
Level of education			
Less than high school	35.6	1.8	<0.001∗
High school graduate or equivalent	25.3	10.7	
Some college	25.3	23.2	
Bachelor's degree	5.7	36.6	
Professional or graduate degree	8.0	27.7	

*Significant findings.

**Table 2:** Odds of support (95% confidence interval) for policy interventions.

Dependent variable: support for obesity prevention policy								
	Tax junk foods and use the funds gained to support the production and distribution of healthy food		Ban fast food vendors in schools		Establish and mandate school lunch nutrition standards		Tax soda/pop	
	White	Latino	White	Latino	White	Latino	White	Latino
Perception of magnitude of obesity problem								
Big	1.35 (0.25, 7.35)	2.60 (0.87, 7.83)	6.48∗ (1.12, 37.62)	4.17∗∗ (1.47, 11.80)	12.59∗∗ (2.21, 71.71)	6.03∗∗∗ (2.01, 18.08)	1.95 (0.36, 10.45)	4.00∗ (1.24, 12.86)
Neutral	0.52 (0.07, 3.66)	1.44 (0.35, 5.91)	2.85 (0.42, 19.62)	1.10 (0.29, 4.21)	4.04 (0.58, 28.08)	1.54 (0.40, 6.04)	0.58 (0.08, 4.04)	0.92 (0.19, 4.48)
Not big (ref)								
Age	0.96 (0.83, 1.11)	1.15 (0.89, 1.49)	1.25∗ (1.05, 1.48)	1.29 (0.96, 1.72)	1.02 (0.83, 1.27)	1.54∗ (1.04, 2.28)	1.00 (0.86, 1.15)	1.13 (0.87, 1.46)
Female	1.19 (0.74, 1.92)	1.25 (0.68, 2.28)	1.35 (0.79, 2.32)	1.93∗ (1.01, 3.68)	1.98∗ (1.02, 3.86)	1.04 (0.47, 2.32)	1.19 (0.75, 1.90)	1.36 (0.73, 2.54)
Education	1.42∗∗ (1.13, 1.78)	1.07 (0.85, 1.35)	1.37∗ (1.07, 1.75)	1.03 (0.80, 1.34)	1.67∗∗∗ (1.23, 2.28)	1.54∗ (1.07, 2.23)	1.24∗ (1.00, 1.54)	1.04 (0.82, 1.32)

Dependent variable: willingness to pay increased taxes for select policy								
	Better public transportation		Increased opportunities for physical activity		Better housing for the poor		More nutritious healthier school lunches	
	White	Latino	White	Latino	White	Latino	White	Latino

Perception of magnitude of obesity problem								
Big	1.67 (0.31, 9.08)	2.25 (0.75, 6.73)	4.52 (0.83, 24.69)	2.71 (0.94, 7.76)	1.47 (0.27, 8.07)	2.98∗ (1.04, 8.55)	4.50 (0.82, 24.69)	4.47∗∗ (1.59, 12.55)
Neutral	1.27 (0.19, 8.35)	1.26 (0.30, 5.35)	2.01 (0.31, 13.11)	1.24 (0.31, 4.97)	0.76 (0.11, 5.18)	3.56 (0.85, 14.84)	1.55 (0.24, 10.18)	1.72 (0.44, 6.76)
Not big (ref)								
Age	1.07 (0.93, 1.24)	1.59∗∗∗ (1.21, 2.08)	0.98 (0.84, 1.15)	1.27 (0.94, 1.73)	1.09 (0.94, 1.27)	1.46∗ (1.10, 1.94)	0.98 (0.83, 1.15)	1.11 (0.79, 1.54)
Female	1.36 (0.85, 2.17)	1.10 (0.59, 2.07)	1.40 (0.85, 2.31)	1.36 (0.68, 2.72)	2.06∗∗ (1.28, 3.32)	0.86 (0.45, 1.65)	2.08∗∗ (1.26, 3.43)	1.74 (0.82, 3.67)
Education	1.52∗∗∗ (1.22, 1.90)	1.09 (0.86, 1.38)	1.52∗∗∗ (1.21, 1.92)	1.34 (1.00, 1.81)	1.42∗∗ (1.14, 1.77)	0.84 (0.66, 1.07)	1.28∗ (1.01, 1.62)	1.09 (0.80, 1.48)

*P value < 0.05, **P value < 0.01, ***P value < 0.001.
